# The Effectiveness of Aqueous Propolis Extract in Reducing the *Enterococcus faecalis* Count in Primary Teeth: An In Vitro Study

**DOI:** 10.1155/ijod/7629615

**Published:** 2025-04-04

**Authors:** Majd Refaay, MHD. Bashier Almonakel, Samar Alsalameh, Ibraheem Hawary, Yasser Alsayed Tolibah

**Affiliations:** ^1^Department of Pediatric Dentistry, Faculty of Dentistry, Damascus University, Damascus, Syria; ^2^Department of Biology, Faculty of Dentistry, Damascus University, Damascus, Syria; ^3^Department of Applied Chemistry Faculty of Chemistry, Damascus University, Damascus, Syria

**Keywords:** aqueous propolis extract, *Enterococcus faecalis*, pediatric endodontics, sodium hypochlorite

## Abstract

**Objective:** To evaluate the efficacy of 11% aqueous propolis extract in eliminating *Enterococcus faecalis* in necrotic pulp canals of primary anterior teeth compared to 2.5% sodium hypochlorite.

**Materials** and Methods: *E. faecalis* were isolated from necrotic primary anterior teeth with periapical lesions, cultured, and incubated using paper points. The research sample comprised 30 extracted single-rooted necrotic primary anterior teeth, divided equally into two groups according to the irrigants used. Access cavities were prepared, and working lengths were determined. Afterward, canals were shaped using K-files, contaminated with *E. faecalis*, and placed in an incubator for a week. Initial microbial swabs were taken, and then each canal was irrigated with either 3 mL of a hand-made 11% aqueous propolis extract or 3 mL of 2.5% sodium hypochlorite for 5 min. Postirrigation microbial swabs were taken, cultured on blood agar plates, and incubated at 37°C for 48 h, followed by colony counts. Statistical tests included the paired sample *T*-test, Wilcoxon signed ranks, and Mann–Whitney *U* tests. The significance level was set at *α* = 0.05.

**Results:** In total, 11% aqueous propolis extract contributed to a 61.8% reduction in *E. faecalis* (*p* < 0.001), while 2.5% sodium hypochlorite contributed to an 84.1% reduction (*p* < 0.001). The average change in logarithmic values in the sodium hypochlorite group was more significant than in the propolis group (*p*=0.002).

**Conclusion:** In total, 11% aqueous propolis extract is antimicrobial against *E. faecalis*. However, its efficacy was less than 2.5% sodium hypochlorite.

## 1. Introduction

Preserving primary teeth is paramount in dentistry to avoid problems resulting from early loss, such as dental crowding, tooth overlap, and nonfunctional oral habits [1]. Primary teeth are equally important as permanent teeth in the natural development of occlusion, arch length, and masticatory function [2].

A pulpectomy is necessary to preserve primary teeth in cases of irreversible pulpitis or pulp necrosis, involving the complete removal of the pulp tissue. The pulpectomy success depends on a combination of appropriate mechanical preparation tools, irrigation, and filling materials. However, the complex anatomy of the root canal system in primary teeth makes complete cleaning difficult [3, 4]. Hence, the importance of irrigant choice in primary teeth had a crucial role due to the complexity of the root canal system, including numerous lateral canals and their proximity to permanent tooth buds [4].

*Enterococcus faecalis*, a bacterium commonly isolated from necrotic root canals, is associated with chronic untreated periapical lesions. It can survive in the root canal even after preparation and irrigation procedures and is frequently isolated in secondary infections in both primary and permanent teeth [4, 5]. Its microbial resistance is attributed to several factors, including fats and fatty acids in its cell membrane and its ability to grow over a wide range of pH levels, with optimal growth at pH 7.5 [6].

Several irrigants have been used to eliminate these bacteria, and sodium hypochlorite was among the most popular. However, its drawbacks, such as unpleasant taste and potential damage if extruded beyond the apex [7, 8], have led to the search for irrigants that combine its cleansing properties while avoiding its disadvantages.

Numerous studies have confirmed the antimicrobial properties of propolis's ethanolic extract against bacteria, viruses, and fungi, considering it biocompatible and nontoxic [9–11]. Propolis also exhibits antimicrobial efficacy against bacteria, including *E. faecalis* [12].

In a previous in vitro pilot study [13], it was found that dissolving propolis tablets in warm sterile distilled water resulted in a final solution of unknown concentration, demonstrated efficacy comparable to 2.5% sodium hypochlorite in reducing the total bacterial count in necrotic root canals. Therefore, this study evaluated 11% hand-made aqueous propolis extract as an irrigant in vitro efficacy against freshly isolated *E. faecalis* in the primary anterior teeth canals.

## 2. Materials and Methods

### 2.1. Study Design and Settings

A controlled comparative in vitro bacterial study evaluated the antimicrobial efficacy of 11% aqueous propolis extract compared to 2.5% sodium hypochlorite solution against *E. faecalis* when treating necrotic primary anterior teeth canals. This study was undertaken from March 2023 to March 2023 at the Department of Pediatric Dentistry of the Faculty of Dentistry, Damascus University, Damascus, Syria. This study adhered to the ethical guidelines of the Declaration of Helsinki and received ethical approval from the Local Research Ethics Committee of the Faculty of Dentistry (approval no. UDDS-375-14122023/SRC-1550).

### 2.2. Sample Size Calculation

Based on the data of a previous study [14], the sample size in the present study was calculated by G^*⁣*^*∗*^^ Power 3.0.10 (Heinrich-Heine-Universität, Düsseldorf, Germany). In the ANOVA study, sample sizes of 15 and 15 were obtained from the two groups. The total sample of 30 subjects achieves 0.55 effect size *f* and 80% power to detect differences with a 0.05 significance level.

### 2.3. Eleven Percent Aqueous Propolis Extract Hand Preparation

The propolis (Chadi Pharma, Damascus, Syria) was cleaned manually. It was immersed in a 4% apple vinegar solution for a day, followed by immersion in the distilled water for another day. Afterward, the aqueous extract was prepared by placing the propolis in an ultrasonic bath at a variable frequency at a temperature of 50°C in a distilled water medium. The propolis extract was then diluted with distilled water to obtain a concentration of 11%. No alcoholic solvents were used at any stage of the extraction process.

### 2.4. *E. faecalis* Isolation

There were no available stored preisolated *E. faecalis* colonies, so they were isolated from an infected necrotic root canal in a central primary incisor with the periapical lesion. A paper point (Gabadent, Guangdong, China) was inserted into the canal and left for 60 s. The swabbing procedure was repeated three times to ensure an accurate clinical representation. The swabs were placed directly into a nutrient medium (Trypticase Soy Broth) and in an incubator at the Technical Institute of Medical Sciences, Faculty of Medicine–University of Damascus, in an aerobic environment at 37°C for 24 h. After observing bacterial growth in the nutrient broth, it was cultured on blood agar plates.

Diagnostic tests were then performed to confirm the identity of the *E. faecalis*. Initially, general tests such as the catalase test were conducted to determine whether the bacteria were cocci or bacilli [15]. Gram staining was also performed to differentiate between Gram-positive and Gram-negative bacteria. After confirming that the bacteria were Gram-positive cocci, selective tests were conducted. The cultures were plated on Bile Esculine Azide Agar (BEAA), a selective medium for *E. faecalis*. Furthermore, a salt tolerance test was conducted by observing bacterial growth on Chapman agar, Mannitol Salt Agar (MSA) [16], which contains mannitol and salt.

Additionally, bacterial identification was performed using the Phoenix Automated System (BD, Ottawa, Canada) [17]. After confirming the bacterial strain's identity, it was preserved on a slant agar slope from Chapman agar medium (HiMedia Mannitol Salt Agar Base, Maharashtra, India) and placed in an incubator for 24 h. Subsequently, it was stored in a refrigerator at 4°C.

### 2.5. Sample Selection, Preparation, Contamination, and the Initial Swabs

This study involved 30 freshly extracted, primary intacted single-rooted human maxillary incisors that were extracted due to prolonged retention. The parents have provided informed consent that their children's extracted teeth will undergo in vitro study. The exclusion criteria were pathological root resorption involving more than one-third of the root, calcified canals, previous endodontic treatment, and fractures or cracks.

The included teeth were thoroughly washed with running water, and then the root surface was cleaned using K-files (Perfect, Guangdong, China) to remove any residual pulp or ligament remnants. Subsequently, they were transferred to plastic vials containing 0.9% saline solution and stored in the refrigerator at 4°C to avoid the effects of the storage medium [18]. Afterward, the crowns were sectioned using diamond disks on a straight handpiece [14].

Then, the pulp chamber was opened, and the working length was determined using the direct method (until the tip of the K-file was visible at the apex of the root). Subsequently, the canals were prepared using K-files based on the working length.

After determining the master apical file, preparation was carried out with three subsequent sizes, with irrigation of the root canal after each instrument during preparation with 1 mL of sodium hypochlorite solution (Septa, Damascus, Syria) using 27-gauge irrigation needles. Upon completion of the preparation, each root canal was irrigated with 5 mL of 2.5% sodium hypochlorite, followed by rinsing with 5 mL of distilled water to ensure debris removal, and then drying with paper points was performed.

The apical area was etched with 37% phosphoric acid (Ultradent, South Jordan, Utah, USA), and the bonding agent (FGM, Fort Lauderdale, Florida, USA) was applied, followed by light curing for 20 s. The apex was then sealed using a flowable composite resin (FGM, Fort Lauderdale, Florida, USA), and the roots were coated with two layers of red nail varnish to prevent leakage of irrigation fluids, in addition to simulating the clinical scenario with an apical seal [19].

The teeth were placed in acrylic molds to facilitate handling during the experiment. Then, a cotton pellet was placed in the apex of each root canal and sealed with temporary restoration. Once the acrylic had fully hardened, the temporary restorations were removed, and the samples were individually placed into autoclave bags for sterilization using moist heat (JSR, Chungchungnam-Do, South Korea) at 121°C for 20 min. After sterilization, three random samples were taken from the total sample, and microbial swabs were taken to confirm sample sterility and negative culture growth (the three samples showed negative culture growth). A bacterial swab was taken using a sterilized loop from the Chapman culture medium and added to the nutrient broth, which was then thoroughly mixed to obtain bacterial suspension. Afterward, each root canal was injected with 50 μL of bacterial suspension using a micropipette (Human, Wiesbaden, Germany) [20]. The teeth were then immersed in brain heart infusion broth. Subsequently, the teeth were placed in an incubator at 37°C (Bacteriological Incubator 6640-01-071-6596/National Appliance Heinicke Co. Tualatin, USA) and left for a week to allow for the formation of a bacterial biofilm to simulate the clinical scenario [20]. After the incubation period, the initial bacterial swab was taken from each root canal, where the canals were filled with sterilized saline solution. A sterile Hedstrom file (Perfect, Guangdong, China) was done in-and-out motion along the root canal dentine. Subsequently, a suitable paper point was used to take the swab. The paper point was inserted to the entire working length and left for 60 s to ensure a clinical representation of the canal [21]. Afterward, the swab was transferred to a sterile Eppendorf tube containing 1 mL of sterilized saline solution. This process was repeated three times to obtain an accurate bacterial representation of the canal. The Eppendorf tube (Seal-Rite Scientific, Inc., Ocala, FL, USA) containing the paper cones was vortexed for 1 min using a vortex device (MIX-28 Touch fairy Vortexer, Frankfurt, Germany) to ensure homogeneity of the solution.

### 2.6. Irrigation Protocol, Taking the Final Swabs, and Bacteria Colony Counting

Group 1 (11% hand-made aquas propolis extract): Root canals were irrigated with 3 mL for 5 min [22].

Group 2 (2.5% sodium hypochlorite solution) (Septa, Damascus, Syria): The root canals were irrigated for 5 min with a 3 mL volume [22].

After irrigation, the root canals were dried along their length with paper points. Then, the bacterial swabs were taken according to the method described for initial sampling, cultured on Petri dishes (using brain heart infusion agar), and placed in an incubator [16]. The plates were then incubated at 37°C for 48 h [20]. Afterward, the plates were removed from the incubator, and the bacterial colonies were counted.

The bacteria colonies were calculated using the following equation:

Number of bacteria/mL

 = Number of colonies forming units counted

 × reciprocal of dilution factor × 20.

(The dilution factor adopted was 1 for samples before and 0 for samples after irrigation, according to a pilot study that determined the feasibility of bacterial counting).

The bacteria units' number was converted into logarithmic numbers to facilitate bacterial colony counting and statistical analysis.

### 2.7. Statistical Analysis

The collected data were tabulated and analyzed using SPSS software (SPSS Version 26, IBM SPSS Inc., Chicago, IL, USA). The Shapiro–Wilk revealed that the quantitative measurements showed an abnormal distribution in sodium hypochlorite group data and a normal distribution in aquas propolis extract group data. Therefore, the Mann–Whitney *U* tests were used to assess the mean logarithm of bacterial reduction between groups. The level of significance was set at *α* = 0.05.

## 3. Results

**Figure 1:**
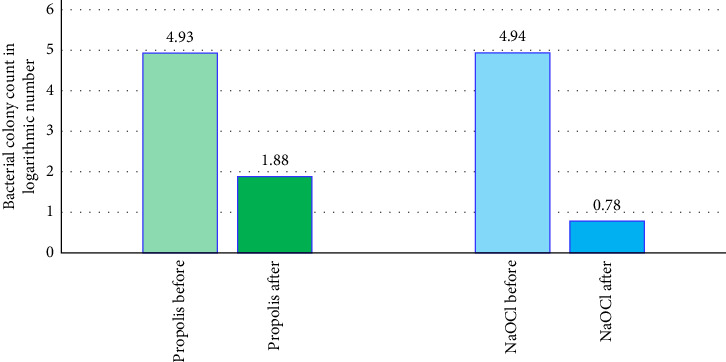
The mean bacterial colony count before and after applying irrigants across groups.

Table 1 and Figure 1 summarize the evaluation of decimal logarithm of the bacterial colony counts among the propolis extract group. *T*-test shows significant difference in 11% hand-made aquas propolis extract group before and after the irrigation process.

From Table 1, it is observed that propolis contributed to the elimination of *E. faecalis* bacteria by 61.8%, with an average reduction of 3.05 log_10_ CFU/mL with a statistically significant difference (*p* < 0.001).

Table 2 and Figure 1 summarize the evaluation of decimal logarithm of the bacterial colony counts among the sodium hypochlorite group. Wilcoxon signed ranks test shows significant difference in sodium hypochlorite group before and after the irrigation process.

From Table 2, it is observed that hypochlorite contributed to a reduction in the count of *E. faecalis* bacteria by an average reduction of 4.16 log_10_ CFU/mL with a statistically significant difference (*p* < 0.001).

A Mann–Whitney *U* test was conducted to assess the significance of the differences in the mean change in logarithmic colony count between the two irrigant groups. Statistically significant differences were observed (*p*=0.002), indicating that hypochlorite was more effective in reducing bacterial counts.

## 4. Discussion

The irrigation process in the primary teeth remains a significant challenge due to the complex anatomy of primary teeth, characterized by numerous lateral canals and high rates of lateral absorption [23, 24].

Sodium hypochlorite solution is commonly used in root canal treatments. Yet, its drawbacks, such as unpleasant taste, chemical burns [6], and poor biocompatibility [25], have spurred the need for an irrigant that combines high antimicrobial efficacy while avoiding these disadvantages. Hence, the effectiveness of 11% aqueous propolis extract as an irrigant was investigated.

The antimicrobial efficacy of propolis is influenced by the type and concentration of the solvent used for extraction and its purification. Aqueous extracts are preferred because they are alcohol-free, resulting in more stable and effective antimicrobial components [26]. Additionally, aqueous propolis extracts exhibit higher biocompatibility than alcoholic extraction [27], where water is considered the most biocompatible solvent for propolis extracts [28], making them suitable for use in primary teeth, where the risk of irrigant leakage into buds is critical. Accordingly, the aqueous medium was adopted in this study.

The 11% concentration of propolis extract was selected based on a pilot study and previous research [21]. Ultrasonic-assisted extraction was adopted because it is effective in extracting bioactive compounds from propolis [29].

*E. faecalis*, a common bacterium found in root canals and associated with chronic untreated apical periodontitis [4], was chosen as the target organism for this study.

Primary anterior teeth were selected due to their susceptibility to early exfoliation and loss [30]. Canal preparation was performed using hand files, whereas a previous study stated no significant difference between manual and mechanical instrumentation [31]. Apex sealing was achieved using a composite resin to prevent irrigant leakage, simulating the clinical scenario of apical seal [19]. Moreover, the teeth were placed in acrylic molds to facilitate handling.

To confirm the identity of *E. faecalis*, various tests were conducted, including catalase testing to determine the bacteria's grouping, Gram staining to distinguish between Gram-positive and Gram-negative bacteria [15], and selective tests, such as culturing on BEAA [32] and salt tolerance testing. BEAA is also a selective medium for these bacteria [16], so it was adopted as a culture medium in the study.

Bacterial swabs were performed using sterilized paper points, following the use of a sterilized hand file with insertion and withdrawal movements along the length of the root canal. As mentioned by Sandini et al. [33], the aim was to obtain a more thorough bacterial swab for the root canal cavities.

The decimal logarithm reduction rate of bacterial count within the root canals was adopted in this study to facilitate statistical analysis and colony counting, as mentioned by researcher Nascimento et al. [34].

The results of this study demonstrated the efficacy of both 11% aqueous propolis extract and 2.5% sodium hypochlorite solution against *E. faecalis*. The antimicrobial properties of propolis stem from several mechanisms. Propolis increases the permeability of the bacterial cell membrane and inhibits the production of adenosine triphosphate (ATP) and ribosomal RNA in bacteria [35]. Additionally, propolis stimulates the formation of insoluble glycans and inhibits the activity of glycosyltransferase enzymes [36], leading to functional and structural damage to bacteria. This occurs through the inhibition of protein formation and disruption of the bacterial cell wall, as well as the prevention of cellular division [37].

This study aligns with the findings of Velani et al. [21] who reported that the ethanolic extract of propolis effectively reduced the count of *E. faecalis* by 85.2%. Arslan et al. [12] and Garg et al. [38] also supported this in their studies, affirming propolis' efficacy against *E. faecalis* when used as an irrigant.

Additionally, the study concurs with Shamma et al. [39], Abbasi et al. [36], and Al-Ostwani, Al-Monaqel, and Al-Tinawi [40] where they demonstrated propolis' effectiveness against *E. faecalis* when used as an intracanal medicament and in combination with zinc oxide and eugenol as an obturation material. On the other hand, Nara et al. [41] showed a lower efficacy of propolis against Enterococcus fecalis. This could be attributed to the type of propolis extract used, as the antimicrobial properties of propolis vary depending on the geographical distribution of the sources from which it is extracted (as mentioned by Tekin and Demirkaya [10]).

The antimicrobial properties of sodium hypochlorite are attributed to its interference with chloramines in cellular metabolism. It also inhibits bacterial enzymes and leads to irreversible oxidation of sulfhydryl groups in essential bacterial enzymes [8].

The superiority of sodium hypochlorite over propolis may be attributed to its possession of chloramines, which result from the reaction between chlorine and amino acids. Chloramines interfere with cell metabolism and inhibit bacterial enzymes, leading to the irreversible oxidation of sulfhydryl groups in key bacterial enzymes, as noted by Nogo-Zivanovic et al. [8]. A previous study met the current results regarding the superiority of sodium hypochlorite solution over propolis extract [42].

Similarly, ethanolic propolis extract and chamomile as irrigants demonstrated effects in reducing the total bacterial count in experimentally infected root canals. However, their efficacy was lower than that of chlorhexidine [11].

On the other hand, Garg et al. [38] and Al Qathami and Madi [13] showed no differences between propolis and sodium hypochlorite solutions against *E. faecalis* biofilm. This may be attributed to differences in the preparation method of propolis and the alcoholic solvent used in propolis extraction, as solvents can affect the bioactive components of propolis.

Additionally, the results differed from a study by Shingare and Chaugule [43], possibly due to differences in working conditions, the quantity, virulence, and type of bacteria isolated from the samples in clinical studies compared to laboratory studies. Moreover, the irrigant composed of nano-sized propolis particles exhibited a similar efficacy to 6% sodium hypochlorite against *E. faecalis* [9].

The limitations of this in vitro study are the one-species biofilm in the applicability of the results for a clinical scenario and the inability to perform a polymerase chain reaction (PCR) test, as PCR plays a crucial role in bacterial identification by providing a rapid, sensitive, and specific method for detecting bacterial DNA.

Further future studies are warranted regarding the aqueous propolis extract, particularly in smear layer removal and its impact on the necrotic root canal biofilm in the clinical situation. Additionally, investigations are needed to assess the influence of irrigation activation systems on enhancing the efficacy of aqueous propolis extracts. These prospective studies will contribute to a better understanding of the benefits of aqueous propolis extract in oral health care and the development of innovative dental treatments. Furthermore, the aqueous propolis extract could be investigated against other bacterial species or a complete biofilm community within root canals. Additionally, studies on its cytotoxicity toward periodontal ligament cells would be essential. A decisive conclusion can be made regarding its clinical application based on comprehensive and rigorous research. While it has demonstrated antimicrobial activity, its effectiveness compared to conventional irrigants needs further evaluation, especially in primary teeth. Given the complex root canal anatomy of primary teeth, which often includes multiple accessory canals, a less effective disinfectant might fail to provide adequate microbial control, potentially compromising treatment outcomes. The authors should discuss whether propolis can be reliably recommended for clinical use and under what conditions its use may be justified.

## 5. Conclusion

In total, 11% aqueous propolis extract exhibits potent antimicrobial activity against *E. faecalis* and can effectively irrigate necrotic primary teeth.

## Figures and Tables

**Table 1:** Descriptive statistics of the decimal logarithm of the bacterial colony counts among the propolis extract group.

Stage	Irrigant	Number	Mean	Standard deviation	Minimum	Maximum	Reduction percentage	*T* value	*⁣* ^*∗*^ *p*-Value
11% hand-made aquas propolis extract	Before applying the irrigant	15	4.93	0.21	4.55	5.35	−61.68%	84.251	**<0.001**
	After applying the irrigant	15	1.88	0.26	1.60	2.34			

*Note*: Bold means that the resulting *p* value is significant (<0.05) (*p* < *α*).

*⁣* ^*∗*^ *T*-test.

**Table 2:** Descriptive statistics of the decimal logarithm of the bacterial colony counts among the sodium hypochlorite group.

Stage	Irrigant	Number	Mean	Standard deviation	Minimum	Maximum	Reduction percentage	*Z* value	*⁣* ^*∗*^ *p*-Value
2.5% sodium hypochlorite	Before applying the irrigant	15	4.94	0.31	4.45	5.50	−84.11%	−3.407771	**<0.001**
	After applying the irrigant	15	0.78	0.78	0.00	1.90			

*Note*: Bold means that the resulting *p* value is significant (<0.05) (*p* < *α*).

*⁣* ^*∗*^Wilcoxon signed ranks test.

## Data Availability

The data that support the findings of this study are available from the corresponding author upon reasonable request.
